# Prognostic factors for ovarian metastases in colorectal cancer patients

**DOI:** 10.1186/s12957-021-02305-3

**Published:** 2021-07-20

**Authors:** Chao Chen, Da Wang, Xiaoxu Ge, Jian Wang, Yuhuai Huang, Tianyi Ling, Tian Jin, Jinhua Yang, Fengping Wang, Weihong Wu, Lifeng Sun

**Affiliations:** 1 Department of Colorectal Surgery and Oncology, Key Laboratory of Cancer Prevention and Intervention, Ministry of Education, the Second Affiliated Hospital, Zhejiang University School of Medicine, 88 Jiefang Road, Hangzhou, Zhejiang Province 310009 People’s Republic of China; 2 Department of Cancer Institute, the Second Affiliated Hospital, Zhejiang University School of Medicine, Hangzhou, Zhejiang Province People’s Republic of China; 3 Department of Gastrointestinal Surgery, Changxing County People’s Hospital, Huzhou, Zhejiang Province People’s Republic of China

**Keywords:** Colorectal cancer, Ovarian metastases, Prognosis factors, Cytoreductive surgery, Scoring system

## Abstract

**Purpose:**

The aim of this study was to analyze prognostic factors for ovarian metastases (OM) in colorectal cancer (CRC) using data from a Chinese center. In addition, the study aimed at developing a new clinical scoring system for prognosis of OM of CRC patients after surgery.

**Patients and methods:**

Data of CRC patients with OM were collected from a single Chinese institution (n = 67). Kaplan-Meier analysis was used to evaluate cumulative survival of patients. Factors associated with prognosis of overall survival (OS) were explored using Cox’s proportional hazard regression models. A scoring system to determine effectiveness of prognosis was developed.

**Results:**

Median OS values for patients with or without surgery were 22 and 7 months, respectively.

Size of OM, number of OM, peritoneal metastasis (PM), Peritoneal cancer index (PCI), and completeness of cytoreduction (CC) were associated with OS of patients through univariate analysis. Multivariate analysis using a Cox regression model showed that only CC was an independent predictor for OS. Three variables (the size of OM >15cm, PCI ≥ 10, and carcinoembryonic antigen (CEA) >30 ng/mL) assigned one point each were used to develop a risk score. The resulting score was used for prognosis of OS.

**Conclusion:**

Surgical treatment of metastatic sites is effective and safe for CRC patients with OM. CC-0 is recommended for improved prognosis. The scoring system developed in this study is effective for prediction of OS of patients after surgery.

**Supplementary Information:**

The online version contains supplementary material available at 10.1186/s12957-021-02305-3.

## Introduction

Colorectal cancer (CRC) is the third most common cancer type in both males and females and the second leading cause of cancer-related death worldwide [1, 2]. Previous studies report that the incidence of ovarian metastasis (OM) in female CRC and in female metastatic CRC are 1.6 (%) ~ 7.2 (%) and 5–10 (%), respectively [3–6]. Approximately 0.6 (%) ~ 4.1 (%) of patients with CRC develop synchronous OM, whereas 0.4 (%) ~ 5.1 (%) of CRC patients develop metachronous OM during disease progression [5, 7, 8]. OM mostly affects young women and develops rapidly. Therefore, patients show symptoms in the later stage. Notably, OM are relatively chemoresistant compared with primary tumors and other metastases [9, 10]. OM are considered end-stage disease and patients receiving palliative chemotherapy have extremely low survival rates (median OS of 10.0 months) [3, 11].

Cytoreductive surgery (CRS) is referred as a therapeutic strategy due to limitations associated with chemotherapy [9, 12–15]. CRS has revolutionized treatment of OM in CRC patients [16]. Patients achieve notable survival benefits (median OS of 36 to 43 months) after undergoing CRS compared with systemic chemotherapy [3]. However, to the best of our knowledge, recurrence, and distant metastasis still exist after detection, and currently, few studies report on the risk stratification and selection of patients who may benefit from surgical oophorectomy. Therefore, it is necessary to develop a clinical criterion for selecting patients to undergo surgical oophorectomy.

Data used in this study were retrieved from a single Chinese center. Data were used evaluate prognostic factors for CRC patients with OM. In addition, a clinical scoring system was developed using pre- and intraoperative factors to predict survival of CRC patients. The findings of this study will provide information and treatment strategies for clinicians and serve as a basis for further research.

## Material and methods

### Ethics and patients

CRC patients (n = 67) presenting with OM from January 2010 to July 2019 were included in this study. Details of surgical oophorectomy were analyzed for 54 patients because 13 patients did not undergo surgery or underwent surgery in a different hospital. The study was approved by the Institutional Review Board of the Second Affiliated Hospital of Zhejiang University School of Medicine. The study was conducted according to the Declaration of Helsinki, Fortaleza, Brazil, 2013. All patients included in the study provided signed informed consent.

### Inclusion and exclusion criteria

Patients were enrolled into the study according to the following criteria: (1) diagnosis of CRC with synchronous or metachronous OM; (2) Eastern Cooperative Group (ECOG) performance status 0 or 1, and no extra-abdominal disease on radiological investigation; and (3) extent of OM evaluated either via contrast-enhanced computed tomography (CT) or magnetic resonance imaging of the ovaries, and treatment was discussed by the multidisciplinary cancer treatment team (MDT). Exclusion criteria were as follows: (1) follow-up time < 12 months from the date of diagnosing OM and (2) extra-abdominal metastasis.

### CRS/HIPEC

Completeness of cytoreduction (CC) was classified as one of four grades (CC-0, -1, -2, and -3) based on the size of residual tumors after CRS. CRS was performed to remove all macroscopic OM or leave lesions < 2.5 mm (CC-0/1), which was considered optimal cytoreduction. Extent of disease was assessed using peritoneal cancer index (PCI) score, as described by Jacquet and Sugarbaker [17]. HIPEC was performed immediately after the abdomen was closed in the operating room. Mitomycin C (30 mg) or oxaliplatin (400 mg) was administered for 60 min at 43 °C in all cases. After postoperative recovery, patients received systemic chemotherapy for a maximum of 24 weeks.

### Clinical follow-up

A follow-up was carried out for all patients in the outpatient unit approximately 2 weeks after treatment, and at least every 3 months for 2 years, then every 6 months after the first 2 years. Carcinoembryonic antigen (CEA), carbohydrate antigen 199 (CA199), and carbohydrate antigen 125 (CA125) markers, and CT scans of the abdomen, pelvis, and thorax, were assessed at each follow-up visit.

### Statistical analysis

Overall survival (OS) was defined as period between the date patients were diagnosed with OM to the last known date of follow-up or date of death. Cumulative survival was evaluated by Kaplan-Meier analysis. Differences in survival curves between groups of patients were assessed using the log-rank test. Multivariate analyses were performed using Cox’s proportional hazard regression models to identify factors associated with OS. A two-sided P value < 0.05 was considered statistically significant. A new clinical scoring system was developed using pre- and intraoperative factors to predict survival of CRC patients. All analyses were performed in IBM SPSS Statistics for Windows, Version 25.0. Armonk, NY: IBM Corp.

## Results

### Clinicopathologic features

**Fig. 1:**
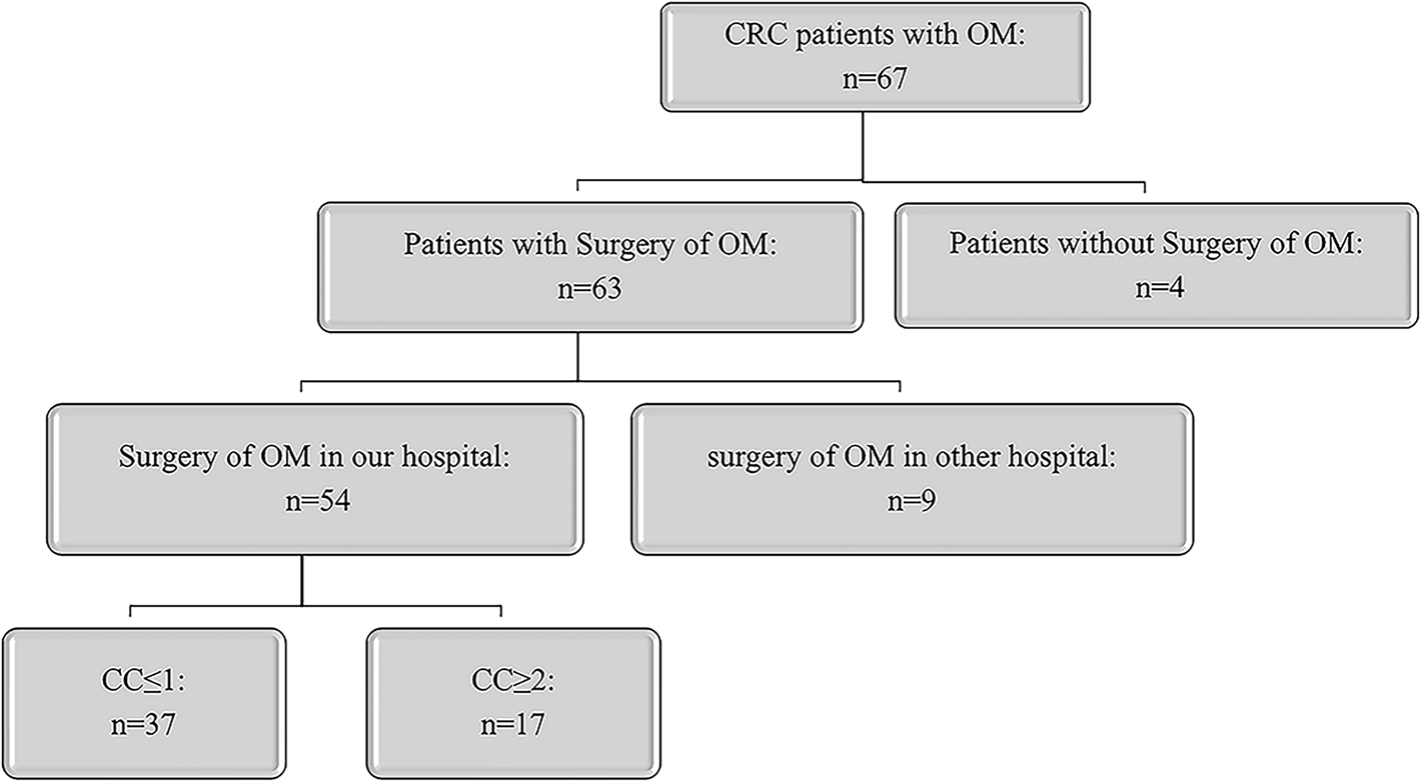
A flowchart of patient included in this study

A total of 67 CRC patients diagnosed with OM between January 2010 and June 2019 in our cancer center were included in this study (Fig. 1). Mean patient age was 49.1 (21–90) years old and 73.1% of the patients were more than 60 years old. Simultaneous OM was found in 53.7% of patients. The most location of primary cancer in CRC patients with OM is left colon (n = 41, 61.2%). Extra-ovarian metastasis occurred in 47 (73.4%) patients and in 35 (54.7%) cases with PM. Adenocarcinoma cancer accounted for 76.1% and patients diagnosed with OM in our center mainly presented with T4 and N1 stage tumors (65.7% and 34.3%, respectively). A total of 27 patients underwent initial surgery for primary tumor in a different center. Surgery for OM was not performed or performed in other hospital for 13 patients, so we analyzed details of surgical oophorectomy for 54 patients; therefore, surgery details were not available for these cases. Therefore, we analyzed surgery data for 54 patients who underwent surgery in our hospital. A total of 32 patients (59.3%) presented with perineural invasion and 22 patients (40.7%) presented with tumor deposits. Most cases (79.6%) presented with lymph node invasion, and the number of lymph nodes invaded was ≥ 4 in 19 patients (35.2%). More than 50% patients presented with PCI ≤ 10 and underwent CC-0/1 in our center. Demographic and histologic data of patients are summarized in Table 1.

**Table 1:** Demographic characteristics of patients

All patients (n=67)		Patients who underwent surgery in our center (n=54)	
Variables	Value, N (%)	Variables	Value, N (%)
Age (year)		HIPEC	
≥60	49 (73.1%)	No	32 (59.3%)
<60	18 (26.9%)	Yes	22 (40.7%)
Primary cancer		Tumor deposits	
Left colon cancer	41 (61.2%)	None	29 (53.7%)
Right colon cancer	24 (35.8%)	Present	10 (18.5%)
Unknown	2 (3.0%)	Unknown	5 (9.3%)
Pathological type		Primary tumor size	
Adenocarcinoma	51 (76.1%)	<5cm	29 (53.7%)
Non-adenocarcinoma	16 (23.9%)	≥5cm	14 (35.9%)
		Unknown	11 (20.4%)
Grade		Perineural invasion	
Grade I	6 (9.0%)	None	17 (31.5%)
Grade II	40 (59.7%)	Present	30 (55.6%)
Grade III	18 (26.9%)	Unknown	7 (13.0%)
Unknown	3 (4.5%)		
T stage		Scope Reg LN Sur	
T0-3	14 (20.9%)	None	13 (24.1%)
T4	44 (65.7%)	Present	36 (66.7%)
Tx	9 (13.4%)	Unknown	5 (9.3%)
N stage		Scope Reg LN Sur (Number)	
N0	15 (22.4%)	<4	32 (59.3%)
N1	23(34.3%)	≥4	17 (31.5% (%))
N2	19 (28.4%)	Unknown	5 (9.3%)
Nx	10 (14.9%)		
Primary tumor size		Metastatic tumor size	
<5cm	30 (44.8%)	≤15cm	37 (68.5%)
≥5cm	15 (22.4%)	>15cm	14 (25.9%)
Unknown	22 (32.8%)	Unknown	3 (5.6%)
Time of OM		Time of OM	
Synchronous	36 (53.7%)	Synchronous	31 (57.4%)
Metachronous	31 (46.3%)	Metachronous	23 (42.6%)
		Number of OM	
		Unilateral	21 (38.9%)
		Bilateral	31 (57.4%)
		Unknown	2 (3.7%)
		Parenchymatous organ metastasis	
		None	18 (33.3%)
		OM+PM	14 (25.9%)
		OM+PM+others	17 (31.5%)
		OM+others	5 (9.3%)
		Peritoneal metastasis	
		No	32 (59.3%)
		Yes	22 (40.7%)
		CA125	
		≤ 40 kU/L	20 (37.0%)
		>40 kU/L	25 (46.3%)
		Unknown	9 (16.7%)
		CEA	
		≤30 mg/L	32 (59.3%)
		>30 mg/L	15 (27.8%)
		Unknown	7 (13.0%)
		CA199	
		≤60 kU/L	31 (57.4%)
		>60 kU/L	16 (29.6%)
		Unknown	7 (13.0%)
		PCI	
		≤10	29 (53.7%)
		>10	25 (46.3%)
		CC	
		0–1	37 (68.5%)
		>1	17 (31.5%)

### Survival outcomes

**Fig. 2:**
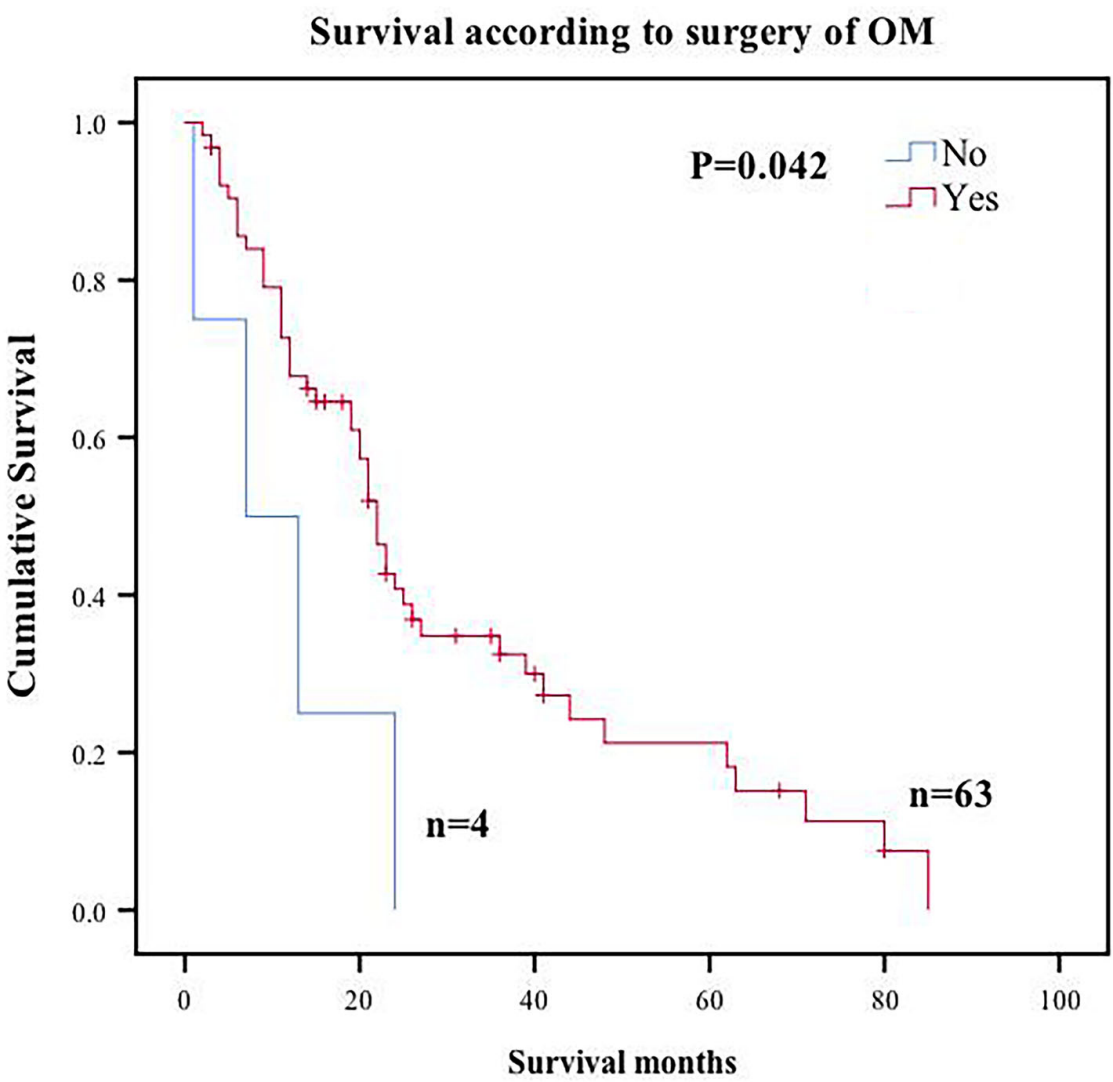
Overall survival Kaplan-Meier curves for surgery of CRC patients with OM

Median follow-up time was 68 (range, 1 to 85) months from the date of OM diagnosis. Median OS for all patients was 22 months, with overall 1- and 3-year survival rates of 66.7% and 30.4%, respectively. A total of 4 patients rejected surgery after OM diagnosis. Median OS for the 4 patients was 7 months compared with 22 months of patients who underwent CRS (Fig. 2).

**Fig. 3:**
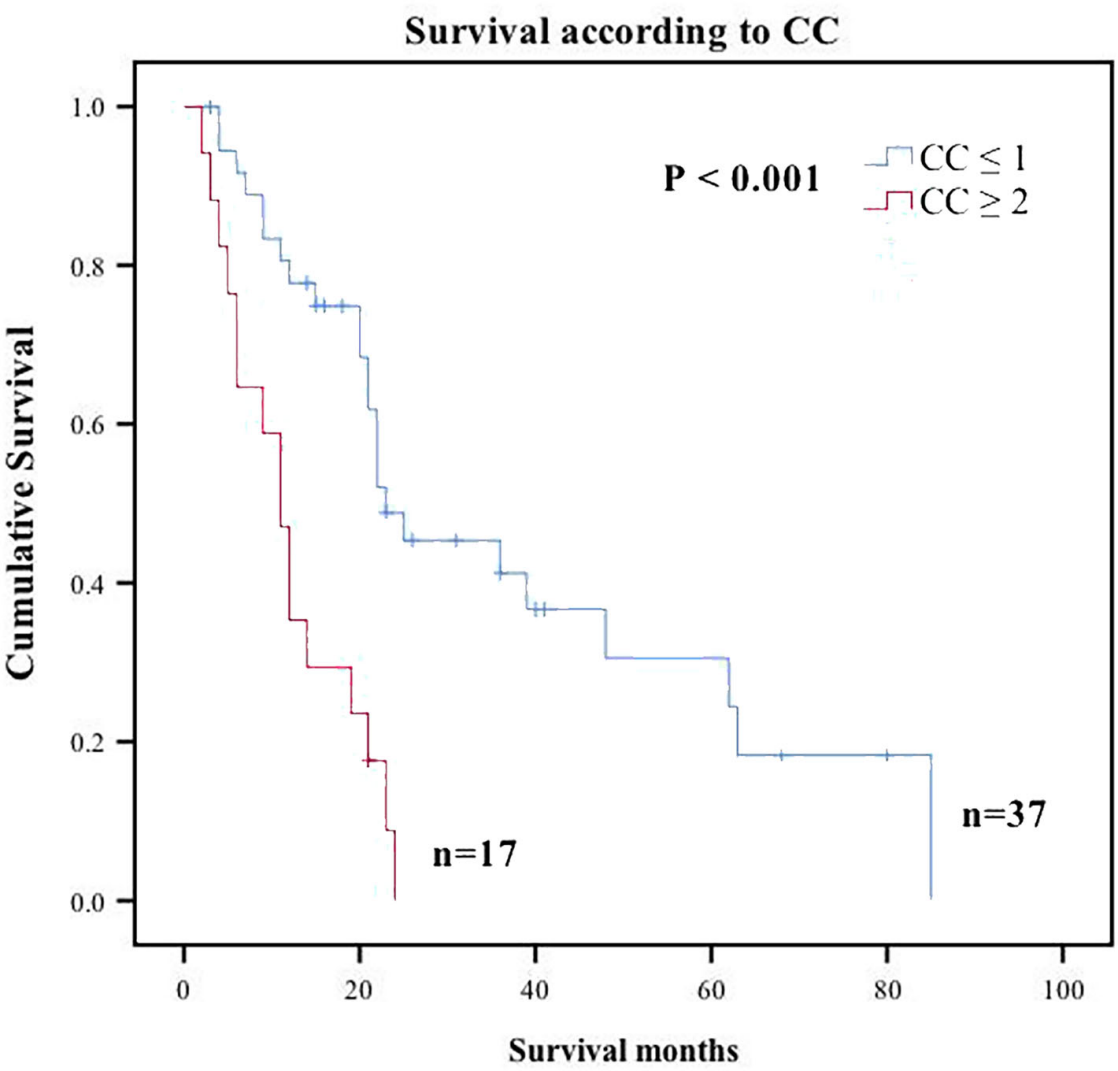
Overall survival Kaplan-Meier curves for significant prognostic variables for CRC patients with OM who underwent surgery. CC, completeness of cytoreduction

Analysis of predictors using Kaplan-Meier method showed that size of OM (P = 0.018), presence of PM (P=0.016), PCI (P = 0.003), and CC score (P < 0.001) were significantly associated with OS (Table 2). However, vascular invasion, perineural invasion, CEA, CA125, or the number of lymph node invasion were not correlated with survival time. In addition, demographic and histologic data, including age, T stage, N stage, grade, pathological subtype, and primary cancer site, were not significantly correlated with survival time (Supplement Table 1). Factors with P value less 0.1 were used for multivariable analysis, and only incomplete cytoreduction was identified as an independent predictor for poor OS (CC >1; HR, 3.782, 95% CI, 1.873 to 7.637; P <0.001) (Table 2, Fig. 3).

**Table 2:** Univariate analysis and multivariate analysis of factors associated with OS using a Cox regression model for patients underwent CRS in our center. Statistically significant P values are presented in bold-italics. P values that are not statistically significant are presented in in italics

Variables	Univariate analysis		Multivariate analysis	
	95%CI	P value	95%CI	P value
Tumor deposits				
None				
Present	1.131 (0.589–2.172)	P=0.711		
Unknown	0.355 (0.081–1.556)	P=0.169		
Perineural invasion				
None				
Present	1.233 (0.613–2.480)	P=0.556		
Unknown	0.375 (0.114–1.234)	P=0.107		
Scope Reg LN Sur				
None				
Present	1.785 (0.781–4.079)	P=0.169		
Unknown	0.515 (0.105–2.535)	P=0.414		
Scope Reg LN Sur (Number)				
<4				
≥4	1.536 (0.792–2.978)	P=0.204		
Unknown	0.394 (0.090–1.724)	P=0.216		
HIPEC				
No				
Yes	0.868 (0.479–1.573)	P=0.641		
Metastatic tumor size				
≤15cm				
>15cm	2.536 (1.174–5.480)	***P=0.018***		
Unknown	0.711 (0.167–3.024)	P=0.644		
Number of OM				
Unilateral				
Bilateral	1.956 (0.965–3.967)	P=0.063		
Unknown	1.779 (0.394–8.021)	P=0.454		
PM				
No				
Yes	2.295 (1.170–4.503)	***P=0.016***		
PCI				
≤10				
>10	2.807 (1.421–5.544)	***P=0.003***		
CA125				
≤ 40 kU/L				
>40 kU/L	1.212 (0.603–2.436)	P=0.589		
unknown	1.012 (0.411–2.491)	P=0.979		
CEA				
≤30 mg/L				
>30 mg/L	0.899 (0.437–1.852)	P=0.773		
unknown	0.728 (0.276–1.920)	P=0.521		
CA199				
≤60 kU/L				
>60 kU/L	0.837 (0.397–1.765)	P=0.641		
unknown	0.713 (0.271–1.876)	P=0.494		
CC				
0				
1	7.412 (2.170–25.317)	***P=0.001***	7.412(2.170-25.317)	***P=0.001***
2	4.827 (2.235–10.422)	***P<0.001***	4.827(2.235-10.422)	***P<0.001***
3	143.854 (8.430–2454.725)	***P=0.001***	143.854(8.430-2454.725)	***P=0.001***
CC				
0–1				
>1	3.782 (1.873–7.637)	***P<0.001***	3.782(1.873-7.637)	***P<0.001***

*Abbreviations: N*, number; *CEA*, carcinoembryonic antigen; *Scope Reg LN Sur*, regional lymph node surgery in surgery; *OS*, overall survival; *HR*, hazard ratio; *CI*, confidence interval

### A new clinical risk score for selecting suitable OM

A new clinical risk score was developed using significant indicators for OS in Kaplan-Meier method including PCI and size of OM. CEA which is important for CRC was also included. Although progression of disease at the level of CC was an independent predictor of prognosis as shown by multivariate analysis, not all patients received surgery. Furthermore, addition of this factor into the risk score model did not improve its prognostic value; therefore, it was omitted from the final model. The score for the corresponding indicators HR value was rounded up to the integer value.

**Fig. 4:**
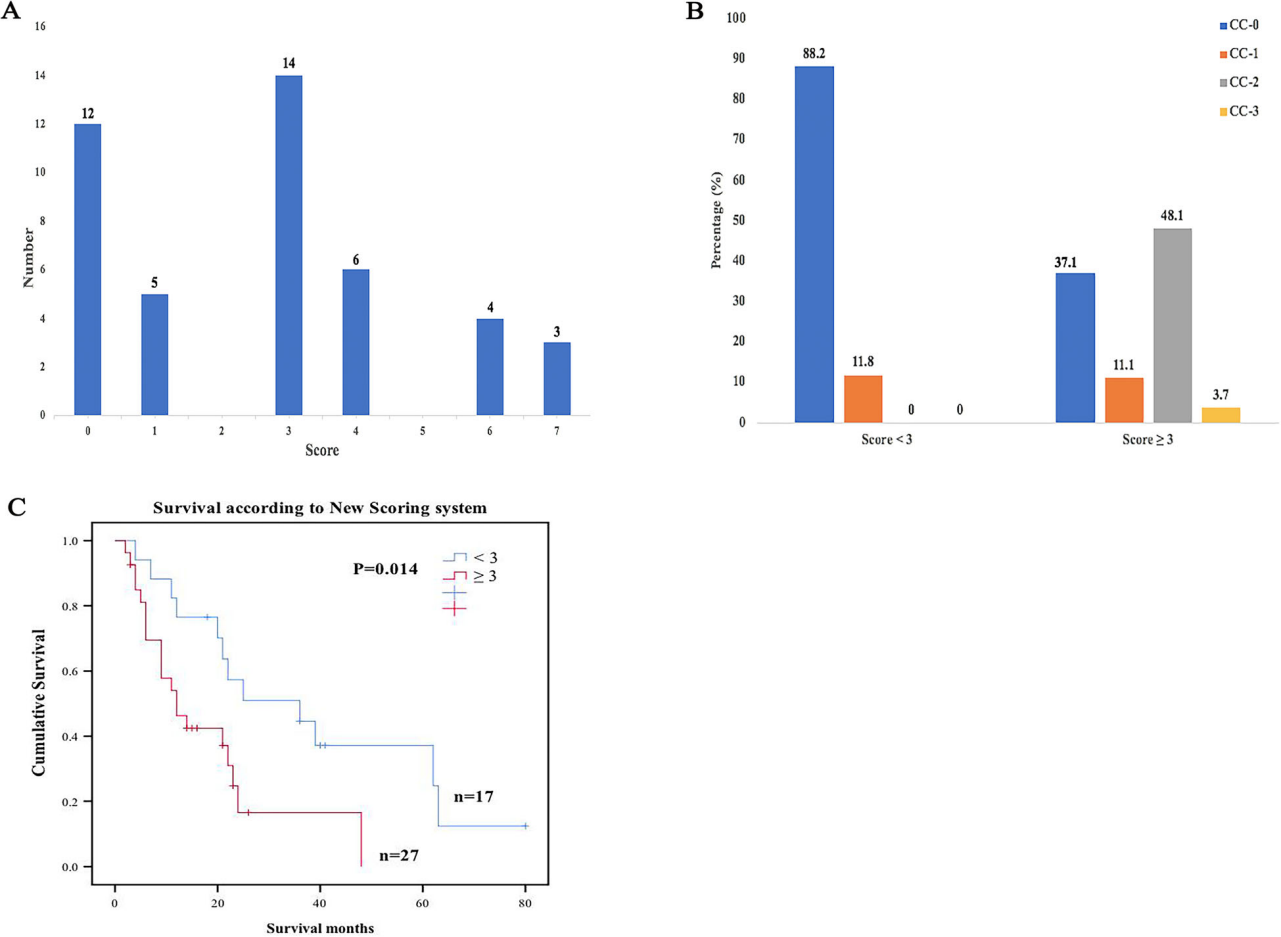
New clinical risk score. **A** Score distribution. **B** Relationship of score and CC. **C** Survival curves based on the new clinical risk score. OS rate were estimated using Kaplan-Meier method with the log-rank test. OS, overall survival

Clinical risk score of all patients was calculated using complete data. The new clinical risk score in patients was calculated with the actual distribution from 0 to 7 points and a median of 3 points and a mode of 3 points (Fig. 4A). Patients were divided into < 3 groups and acuity grouping for subsequent analysis using cut-off value of 3 points (median). A score < 3 patients resulted in a high CC-0 ratio (88.2%), and most patients with a score ≥ 3 points did not reach tumor removal stage (Fig. 4B). A high score was positively correlated with poor overall survival. Patients who scored <3 (low risk) had 1-, 3-, and 5-year survival of 76.5%, 44.6%, and 37.2%, respectively, and median survival of 36 months. Patients who scored ≥ 3 (high risk) had a 3-year survival of 16.5% with no survivors beyond 5 years and median survival of 12 months (Fig. 4C).

## Discussion

Previous studies have explored factors associated with prognosis of CRC patients with OM. However, this is the first study to develop a new clinical risk score to help in preoperative or intraoperative decision making. OM affects young women, develops rapidly, and is relatively chemoresistant; therefore, there is a need to develop effective treatment of OM patients [9]. Previous studies have reported controversial results on CRS for CRC patients with OM. Some surgeons recommend CRS as it improves CRC patient survival [16, 18–20]. However, some studies report that CRS approach is ineffective [21, 22]. In this study, median OS for CRC patients with OM group who underwent surgery was 22 months, compared with median OS of 10 months for patients receiving palliative treatment reported by Lee et al. [11]. Previous studies report that CRS affects long-term prognosis and recurrence of patients with CRC [23–29] and similar results were reported in our study. CRC patients with OM who achieved CC-0 showed a median OS of 36 months, whereas patients who did not achieve CC-0 showed a median OS of 3 months. Multivariate analysis showed that incomplete cytoreduction is an independent risk factor for OS. However, HIPEC was not associated with OS of CRC patients in our study, which can be attributed to the small sample size. The results of the current study show that CRS should be performed on CRC patients as it is safe, feasible, and effective for treatment of diverse advanced tumors.

However, some researches showed the influence of ovariectomy for female [30–32]. Ovariectomy will cause menopause in young patients, which makes a sudden perimenopausal syndrome, and the more severe symptoms than natural [30]. Besides, a cohort study in Britain reported that early menopause is a risk of ischemic stroke (early menopause vs natural menopause, HR = 1. 5, 95% CI 1. 01~2. 25) [31]. According to Mayo Clinic Oophorectomy and Aging Cohort Study, early menopause caused by surgery is associated with osteoporosis, worse neurocognitive performance, and symptoms of depression or anxiety [32, 33].

The findings of this study show that complete resection of ovarian metastasis is positively correlated with a better prognosis. However, CRC patients with OM to undergo surgery should be selected carefully. Preoperative assessment of suitable patients for aggressive treatment mode can reduce switching operation rate, incomplete tumor surgery rate, and perioperative mortality. Currently, there is no unified standard but some considerations include informed consent and will of patient, ECOG < 2, no serious complications, acceptable quality of life, asymptomatic, lack of tumor progression during chemotherapy, absence of extravasation, resectable liver metastases <= 3, intestinal stenosis <= 1, no widespread intestinal disease, no biliary or ureteral obstruction, stomach liver toughening with involvement < 5 cm, no mesenteric root or pancreatic infiltration, possibility of completing CC0-1, and PCI < 20; however, prognosis of patients are different. Several prognostic scoring system have been reported as references for CRC patients with PM, including Colorectal Peritoneal Score [34], Peritoneal Surface Disease Severity Score (PSDSS) [35], and Colorectal Peritoneal Metastases Prognostic Surgical Score [36, 37]. Pelz et al. developed a PSDSS based on clinical symptoms, PCI, and histology to serve as a prognostic tool for overall survival for clinicians and researchers. Simkens et al. evaluated peritoneal surface disease PSDSS and suggested COMPASS, including age, PCI, locoregional lymph node status, and signet ring cell histology. However, no other single factor examined reliably differentiated suitable patients to undergo surgery from CRC patients with OM who met an early demise. Therefore, we sought to develop a scoring system using multiple factors to provide information on pre-operative or intra-operative decision making. The alternative system uses 3 variables including PCI, size of OM, and CEA > 30ng/mL. In order to maximize clinical utility, only variables known preoperatively and/or intraoperatively were included. Therefore, although completeness of OM resection was prognostic factor for survival as shown by multivariate analysis, CC was not used in building our predictive model. All three factors in the final scoring model were weighted based on comparable hazard ratios (2.295, 2.536, and 0.899 for PCI, size of OM and CEA > 30ng/mL, respectively).

A group of patients (risk score ≥ 3) who performed poorly after resection were identified using this proposed risk scoring system. These outcomes were consistent with modern chemotherapy outcomes (median OS=12). The findings show that higher score is correlated with poor prognosis. High clinical risk score was associated with significant decrease in proportion of CC-0 patients, whereas the proportion of CC-3 or CC-4 patients was significantly increased. These findings imply that CC-3 or CC-4 patients should not undergo resection. In contrast, patients with < 3 points showed comparable survival to patients with surgery.

Prognostic factors were analyzed and a new clinical risk score for CRC patients with OM was developed using data from our center; however, our study had some limitations. First, this was a retrospective study; therefore, it had potential bias. Second, we used a sample size comprising 67 CRC patients with OM. These limitations could be ameliorated by recruitment of more patients for inclusion in a future prospective study. Studies including more samples should be carried out to assess effectiveness of treatments and explore effective prognostic factors for CRC patients with OM.

## Conclusion

In summary, surgery is an effective and safe treatment approach for CRC patients with OM. In addition, surgery of the metastatic site should be recommended for CRC patients with OM to achieve CC-0. OM should not be considered an absolute contraindication to curative resection; however, appropriate selection is important. The proposed scoring system provides a basis for identification of a subset of patients who do not benefit from resection.

## Supplementary Information

**Additional file 1: Supplemental Table 1.** Univariate analysis and multivariate analysis of factors associated with OS with a cox regression model in all patients. OS, overall survival; HR, Hazard Ratio; CI, Confidence Interval; Statistically significant P values are presented in bold-italics. P values that are not statistically significant are presented in italics.

## Data Availability

All data generated or analyzed during this study are included in this published article.

## Abbreviations

CRC: Colorectal cancer
PM: Peritoneal metastasis
OM: Ovarian metastasis
OS: Overall survival
PCI: Peritoneal cancer index
CRS/HIPEC: Cytoreductive surgery combined with hyperthermic intraperitoneal chemotherapy
CEA: Carcinoembryonic antigen
Reg LN Sur: Regional lymph node surgery in surgery
PNI: Perineural invasion
