# Supplementary material for: Prognostic factors for ovarian metastases in colorectal cancer patients
Source: World J Surg Oncol. 2021 Jul 20;19:220. doi: 10.1186/s12957-021-02305-3 (PMC8293531; doi:10.1186/s12957-021-02305-3)
Supplement: Supplementary file 1 — Additional file 1: Supplemental Table 1. Univariate analysis and multivariate analysis of factors associated with OS with a cox regression model in all patients. OS, overall survival; HR, Hazard Ratio; CI, Confidence Interval; Statistically significant P values are presented in bold-italics. P values that are not statistically significant are presented in italics. [file 12957_2021_2305_MOESM1_ESM.docx]

| **Variables** | univariate analysis | |
| --- | --- | --- |
|  | **95%CI** | **P value** |
| Age (year)  ≥60  <60 | 0.965(0.510-1.828) | P=0.913 |
| Primary cancer  Left Colon cancer  Right colon cancer  Unknown | 1.026(0.570-1.847)  0.000(0-3.330E+277) | P=0.931  P=0.971 |
| Pathological type  Adenocarcinoma  Non- Adenocarcinoma | 1.147(0.619-2.123) | P=0.664 |
| Grade  Grade I  Grade II  Grade III  Unknown | 0.867(0.332-2.265)  1.280(0.466-3.518)  1.893(0.450-7.965) | P=0.770  P=0.632  P=0.384 |
| T stage  T0-3  T4  Tx | 1.314(0.649-2.660)  0.721(0.261-1.993) | P=0.447  P=0.528 |
| N stage  N0  N1  N2  Nx | 1.597(0.714-3.572)  2.149(0.946-4.881)  1.251(0.465-3.365) | P=0.254  P=0.068  P=0.658 |
| Primary Tumor Size  <5cm  ≥5cm  Unknown | 1.610(0.806-3.215)  0.822(0.430-1.571) | P=0.178  P=0.554 |
| Time of OM  synchronous  metachronous | 0.687(0.391-1.207) | P=0.192 |
|  |  |  |

**Note:**

**Abbreviations:** N: number; CEA: carcinoembryonic antigen; Scope Reg LN Sur: Regional Lymph Node Surgery in surgery.
